# Reduction of MLH1 and PMS2 confers temozolomide resistance and is associated with recurrence of glioblastoma

**DOI:** 10.18632/oncotarget.1302

**Published:** 2013-10-14

**Authors:** Yoshinari Shinsato, Tatsuhiko Furukawa, Shunji Yunoue, Hajime Yonezawa, Kentarou Minami, Yukihiko Nishizawa, Ryuji Ikeda, Kohichi Kawahara, Masatatsu Yamamoto, Hirofumi Hirano, Hiroshi Tokimura, Kazunori Arita

**Affiliations:** ^1^ Department of Neurosurgery, Graduate School of Medical and Dental Sciences Kagoshima University, Kagoshima, Japan; ^2^ Department of Molecular Oncology, Graduate School of Medical and Dental Sciences Kagoshima University, Kagoshima, Japan; ^3^ Department of Clinical Pharmacy and Pharmacology, Graduate School of Medical and Dental Sciences Kagoshima University, Kagoshima, Japan

**Keywords:** temozolomide, MLH1, PMS2, MutL-alpha, resistance, recurrence, glioblastoma

## Abstract

Although there is a relationship between DNA repair deficiency and temozolomide (TMZ) resistance in glioblastoma (GBM), it remains unclear which molecule is associated with GBM recurrence. We isolated three TMZ-resistant human GBM cell lines and examined the expression of O^6^-methylguanine-DNA methyltransferase (MGMT) and mismatch repair (MMR) components. We used immunohistochemical analysis to compare MutL homolog 1 (MLH1), postmeiotic segregation increased 2 (PMS2) and MGMT expression in primary and recurrent GBM specimens obtained from GBM patients during TMZ treatment. We found a reduction in MLH1 expression and a subsequent reduction in PMS2 protein levels in TMZ-resistant cells. Furthermore, MLH1 or PMS2 knockdown confered TMZ resistance. In recurrent GBM tumours, the expression of MLH1 and PMS2 was reduced when compared to primary tumours.

## INTRODUCTION

Treatment with the alkylating agent temozolomide (TMZ) has resulted in benefits for patients with glioblastoma (GBM). Nevertheless, almost all GBMs recur and lead to death of the patients [1-3]. Intrinsic or acquired resistance to TMZ, is one of the greatest obstacles in successful GBM treatment, and is thought to be influenced by a variety of mechanisms.

Studies indicate that DNA repair molecule deficiency is linked to the acquisition of TMZ resistance in GBM; however clarification on which molecules are most important in the attainment of TMZ resistance is required. For instance, O^6^-methylguanine-DNA methyltransferase (MGMT) (ENSG00000170430), which is the best-known cause of TMZ resistance, is only expressed at low levels in many GBMs due to the methylation of its promoter region. Mismatch repair (MMR)-dependent correction of replication errors and responses to DNA damage requires heterodimeric complexes of MutS-alpha (mutS homolog 6; MSH6; ENSG00000116062 / mutS homolog 2; MSH2; ENSG00000095002) and MutL-alpha (MutL homolog 1; MLH1; ENSG00000076242 / postmeiotic segregation increased 2; PMS2; ENSG00000122512). MutS-alpha initially recognizes DNA mismatches, while MutL-alpha identifies the mismatch and subsequently excises the nascent error containing DNA strand [4]. Previous studies have suggested that there is a relationship between deficiencies in these four MMR components and GBM recurrence [5-7]; however it remains unclear which MMR components are most important in influencing the acquisition of TMZ resistance.

Therefore, we established TMZ-resistant cell lines that do not express MGMT from U251 human GBM cells and analysed their TMZ resistance. In addition, we analysed the expression of MLH1, PMS2 and MGMT in primary and recurrent GBM tumour samples obtained from patients with GBM recurrence during TMZ treatment.

## RESULTS

### Generation and analysis of TMZ-resistant cell lines

To analyse cellular TMZ resistance mechanisms, we generated TMZ-resistant U251 cell lines (U251/TMZR1, U251/TMZR2 and U251/TMZR3 cells). MTT assays showed that these cells displayed a resistance to TMZ that was increased by 6.7-fold, 12.9-fold and 8.4-fold, respectively, when compared with that of U251 cells. In addition, the TMZ-resistant cells showed cross-resistance to a SN1-type methylating agent, N-Methyl-N ʹ -nitro-N-nitrosoguanidine (MNNG), which has properties similar to those of TMZ. The cells did not show obvious resistance to other types of methylating agents such as nimustine (ACNU), which is a SN2-type methylating agent, or methyl methanesulfonate (MMS), which, unlike TMZ, does not add methyl groups to the O^6^ position of guanine nucleotides (Table 1).

**Table 1:** Sensitivity of TMZ-resistant cells to several methylating reagents

Cells	U251	U251/TMZR1		U251/TMZR2		U251/TMZR3	
Reagent	IC_50_	IC_50_	RR	IC_50_	RR	IC_50_	RR
TMZ (mM)	67.6 ± 11.2	454.1 ± 30.0	6.7 ± 0.9^a^	871.0 ± 105.4	12.9 ± 0.8^a^	569.5 ± 44.7	8.4 ± 0.8^a^
MNNG (ng/ml)	0.45 ± 0.02	2.6 ± 0.04	5.6 ± 0.3^a^	3.5 ± 0.22	7.7 ± 0.7^a^	1.7 ± 0.04	3.8 ± 0.09^a^
ACNU (mM)	87.3 ± 3.8	32.3 ± 1.75	0.37 ± 0.01 ^a^	58.1 ± 4.4	0.7 ± 0.05^a^	111.8 ± 4.7	1.7 ± 0.1^a^
MMS (mM)	131.5 ± 9.1	88.4 ± 4.6	0.7 ± 0.03^a^	206.2 ± 1.6	1.6 ± 0.1^a^	165.0 ± 8.9	1.3 ± 0.1^a^

Cell survival was determined using the MTT assay.

Data are means ± S.D. of three determinations obtained from triplicate cultures.

a Significantly different (p < 0.05) compared with U251 cells, as determined by Student's t test.

U251, parental glioblastoma cell line; TMZR1-R3, TMZ-resistant U251 cells; TMZ: temozolomide; MNNG: N-methyl-N’-nitro-N-nitrosoguanidine; ACNU: Nimustine; MMS: methyl methanesulfonate; RR: relative resistance (fold resistance compared to U251 parental cells).

### MGMT is not involved in the acquisition of resistance to TMZ

An increase in cellular MGMT activity is the best-known mechanism by which cells acquire TMZ resistance [8, 9]. However, neither MGMT mRNA nor MGMT protein were detectable in the TMZ-resistant cells or the U251 cells (Supplementary Figure S1A, B). The *MGMT* promoter region has been reported to be methylated and, thereby, inactivated in U251 cells. Therefore, we examined the status of the promoter region of the *MGMT* gene in U251 and the TMZ-resistant cells by using methylation-specific PCR. This analysis indicated that the promoter region of the *MGMT* gene in U251 and TMZ-resistant cells was methylated (Supplementary Figure S1C).

### G2/M arrest and apoptosis is induced by TMZ in U251 cells but not in U251/TMZR2 cells

Next, the cell cycle populations of U251 and U251/TMZR2 were analysed to determine whether the decreased sensitivity of the U251/TMZR2 cells to TMZ resulted in a reduction of cell cycle arrest and cell death. After treatment with 800 micro-M TMZ for 120 h, U251 cells were mostly arrested in the G2/M phase of the cell cycle, and there was an increase in the sub-G_1_ fraction of cells when compared to the control cells. In contrast, TMZ treatment did not alter the cell cycle distribution, or the sub-G_1_ fraction of U251/TMZR2 cells when compared to control cells (Supplementary Figure S2A). We then measured caspase-3 activity in U251 and U251/TMZR2 cells after treatment with 800 micro M TMZ for 96 h. Our results showed that *caspase*-3 activity in U251 cells was *5.9* ± 0.6*-fold higher than* in U251/TMZR2 cells (*p* < 0.01) (Supplementary Figure S2B). These results demonstrate that TMZ induces MMR mediated G2/M arrest and apoptosis in parental cells, whereas acquired resistance to TMZ protects cells from TMZ-induced G2/M arrest and apoptosis.

### Reduction of MLH1 expression and subsequent reduction in PMS2 protein expression is involved in TMZ resistance

**Figure 1:**
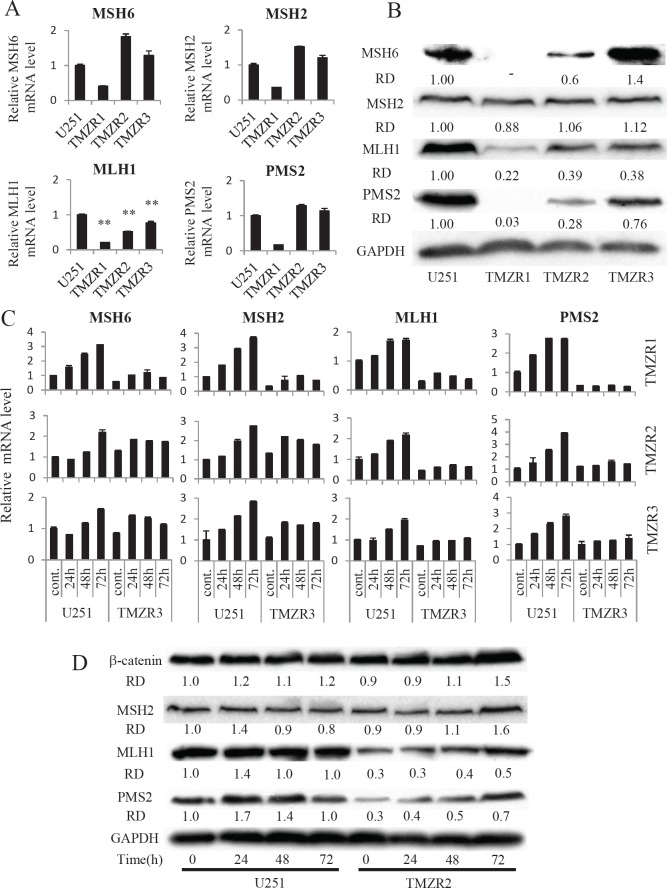
The expression of mismatch repair components in TMZ-resistant cells

DNA alkylating agents such as MNNG and TMZ have been reported to induce MMR, DNA damage-induced G2 checkpoint, and apoptosis [10-14]. To determine whether MMR systems were altered in the TMZ-resistant cells, we compared the expression of the MMR proteins MSH6, MSH2, MLH1, and PMS2 in U251 cells and TMZ-resistant cells. We found that the mRNA and protein expression of MLH1 was consistently lower in the TMZ-resistant cells than in the U251 cells (Fig. 1A, B). Furthermore, the mRNA expression of MLH1 was significantly induced by TMZ in a time dependent manner in U251 cells, whereas only slight TMZ-mediated inductions were observed in the three TMZ-resistant cell lines. In addition, the expression of MLH1 protein in TMZ-resistant cells was also lower than that of U251 cells at all time points after TMZ treatment (Fig. 1C, D). Notably, the expression of PMS2 protein was correlated with the expression of MLH1 protein but not to PMS2 mRNA expression levels in these three TMZ-resistant cell lines. In addition, the induction of PMS2 protein and mRNA, as well as MLH1 protein, after TMZ treatment was also lower than that in the parent cells (Fig. 1C, D). These results suggest that the reduction of MLH1 and/or PMS2 is involved in TMZ resistance.

(A) The mRNA levels of MSH6, MSH2, MLH1, PMS2 in U251 and TMZ-resistant cells (U251/TMZR1,U251/TMZR2 and U251/TMZR3 cells) was analysed using real-time PCR. GAPDH mRNA expression levels were used to normalize the values obtained for each gene. Columns are representative of triplicate independent experiments and bars represent SD. **, *P* < 0.01, significantly different from the value of U251 cells. (B) The level of MSH6, MSH2, MLH1 and PMS2 protein expression in U251 and TMZ-resistant cells was detected by immunoblotting, as described in the Materials and Methods. GAPDH protein levels were assayed as loading controls. The densities of the individual bands were quantified using Alpha View software, and were normalized to GAPDH in order to obtain the relative densities (RD). (C) Time-dependent changes in MMR gene expression after 400 micro-M TMZ treatment at the indicated times were analysed using real-time PCR. GAPDH mRNA expression was used to normalize the values obtained for the MMR genes. (D) Time-dependent changes in MSH2, MLH1 and PMS2 protein expression after 400 micro-M TMZ treatment at the indicated times were detected by immunoblotting as described in the Materials and Methods. beta-Catenin protein levels were used as a negative control. GAPDH protein levels were assayed as loading controls. Densities of the individual bands were quantified using Alpha View software and normalized to GAPDH in order to obtain the relative densities (RD).

### Diminished MLH1 expression reduces the expression of PMS2 protein and modulates TMZ sensitivity in several GBM cell lines

**Figure 2:**
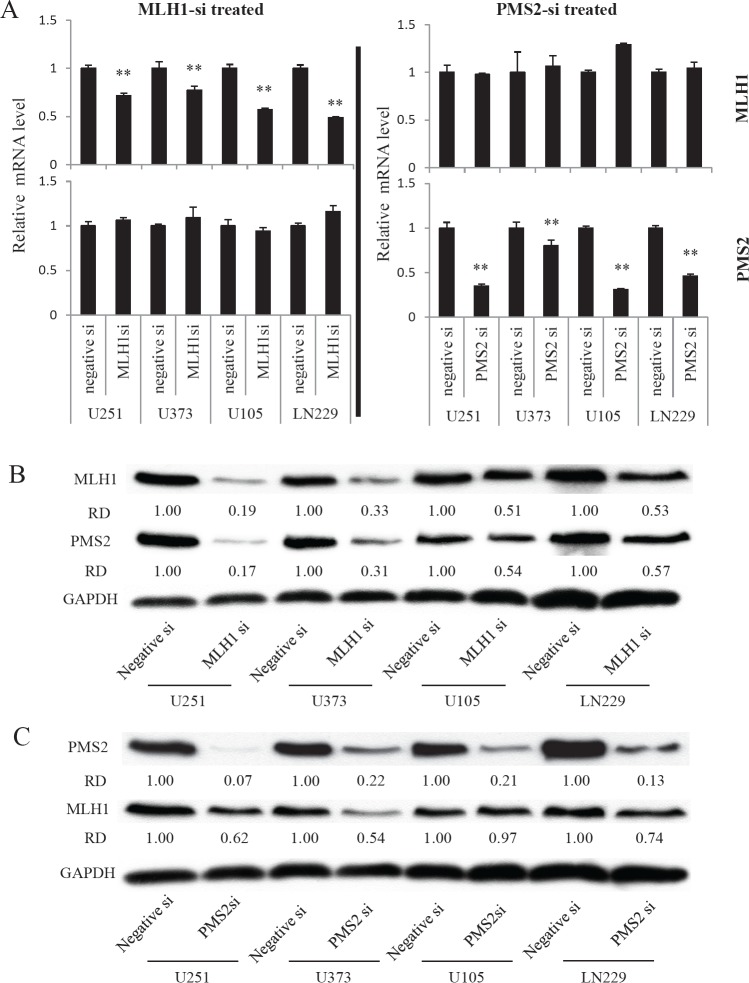
Effect of MLH1 or PMS2-specific siRNA treatment on MLH1 or PMS2 expression in several GBM cell lines

To confirm whether the reduction of MLH1 expression attenuates PMS2 protein and confers TMZ resistance, we tested several GBM cells using MLH1 siRNA knockdown. MLH1-specific siRNA significantly reduced not only the mRNA and protein expression of MLH1 in GBM cells, but also the expression of PMS2 protein (Fig. 2A, B). Notably, knockdown of MLH1 did not affect the expression of PMS2 mRNA (Fig. 2A). In contrast, knockdown of PMS2 by siRNA did not affect the mRNA expression of MLH1, but slightly affected the protein expression of MLH1 (Fig. 2A, C). In addition, MLH1 or PMS2-specific siRNA-treated GBM cells were resistant to TMZ when compared to control siRNA-treated cells (Table 2). These data indicate that MLH1 is involved in PMS2 protein stability, and attenuation of MLH1 and PMS2 confers TMZ resistance to GBM cells.

GBM cells were transfected with 37.5 nM negative control (Negative-si), MLH1-specific (MLH-si), or PMS2-specific (PMS2-si) siRNA. (A) At 48 h, the mRNA levels of MLH1 and PMS2 were analysed using real-time PCR. GAPDH mRNA expression was used to normalize the values obtained for MLH1 and PMS2. Columns are representative of triplicate independent experiments, and bars indicate SD. **, *P* < 0.01, significantly different from Negative-si treated GBM cells. (B) The protein levels of MLH1 or PMS2 in Negative-si or MLH1-si treated GBM cells were detected by immunoblotting, as described in the Materials and Methods. GAPDH protein levels were assayed as the loading control. The densities of the individual bands were quantified using Alpha View software, and normalized to GAPDH in order to obtain the relative densities (RD). (C) The protein levels of MLH1 or PMS2 in Negative-si or PMS2-si treated GBM cells were detected by immunoblotting, as described in the Materials and Methods. GAPDH protein levels were assayed as the loading control. The densities of individual bands were quantified using Alpha View software and normalized to GAPDH in order to obtain relative densities (RD).

**Table 2:** MLH1 and PMS2 expression levels modulate the TMZ sensitivity of several glioma cells

cells	U251		U373		U105		LN229	
	IC_50_^a^(μM)	RR	IC_50_^a^(μM)	RR	IC_50_^a^(μM)	RR	IC_50_^a^(μM)	RR
Negative si	46.9 ± 7.3	−	10.7 ± 1.3	−	116.9 ± 31.6	−	84.9 ± 22.8	−
MLH1 si	529.3 ± 25.6	11.4 ± 1.3 ^b^	125.1 ± 29.3	11.6 ± 1.3 ^b^	641.1 ± 15.6	5.7 ± 1.5 ^b^	175.2 ± 19.1	2.1 ± 0.4 ^b^
PMS2 si	625.6 ± 49.4	13.4 ± 1.1 ^b^	131.5 ± 6.8	12.4 ± 1.1 ^b^	671.4 ± 46.0	6.0 ± 1.2 ^b^	310.0 ± 87.2	3.7 ± 0.1 ^b^

a Cell survival was determined using the MTT assay.

b Significantly different (p < 0.05) compared with Negative siRNA-treated cells, as determined by Student's t test.

### The expression of MLH1 and PMS2 is reduced in recurrent human glioblastomas during administration of TMZ

**Figure 3:**
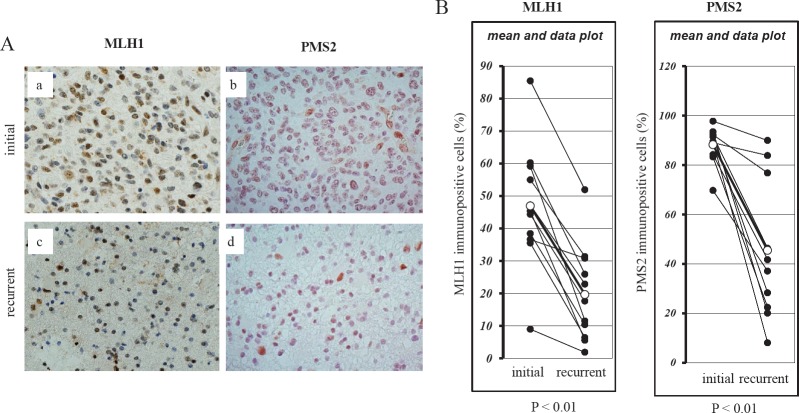
Immunohistochemical analysis of the expression of MLH1 and PMS2 in recurrent human glioblastomas during TMZ treatment

If the reduction of MLH1 and/or PMS2 expression plays an important role in the acquisition of TMZ resistance in *vivo* as well as in *vitro*, MLH1 and/or PMS2 attenuation might be involved in the recurrence of GBMs. We therefore evaluated MLH1 and PMS2 expression in a total of 11 clinical GBM and AA specimens using immunohistochemical methods. Immunohistochemical analysis of initial and recurrent tumours of a representative case that was treated with TMZ is shown in Fig. 3A, a-d For all cases, the expressions of MLH1 and PMS2 were significantly decreased in the recurrent specimens (19.7 ± 14.8; 45.3 ± 27.2%) in comparison to their respective initial tumours (46.9 ± 19.1; 88.0 ± 7.4%) (*p* < 0.01, paired *t*-test) (Fig. 3B). By contrast, there was no correlation between the attenuation of MGMT expression and recurrence (Supplementary Figure S3A, B).

MLH1 and PMS2 protein expression in nine clinical glioblastoma and two anaplastic astrocytoma specimens was analysed by using immunohistochemical analysis, as described in the Materials and Methods. The fraction of MLH1 or PMS2 positive cells in each specimen was determined by dividing the number of MLH1 or PMS2 immunopositive cells by the total cell number in three microscopic fields (magnification × 400). (A) Immunohistochemical staining of initial and recurrent tumours of one representative case. (a) MLH1 staining in the initial tumour (44.3 ± 3.1% positive nuclei) versus (c) the recurrent tumour (17.7 ± 3.8% positive nuclei). (b) PMS2 staining in the initial tumour (92.3 ± 3.5% positive nuclei) versus (d) the recurrent tumour (28.3 ± 6.9% positive nuclei). (B) The percentage of MLH1 and PMS2 immunopositive cells in each clinical initial and recurrent gliobastoma specimens is shown (paired *t*-test, *P* < 0.01).

## DISCUSSION

Previous studies have suggested that MGMT and base excision repair (BER) are involved in TMZ resistance [15, 16]. MGMT also repairs the O^6^-chloroethylguanine residues induced by SN2-type methylating agents such as ACNU [17, 18]. Additionally, BER is involved in MMS resistance [19]. However, our TMZ-resistant cells did not show cross-resistance to such methylating agents (Table1). In addition, the *MGMT* promoter was methylated in both the TMZ-resistant cells as well as the parental U251 cells (Supplementary Figure S1C). These results suggest that MGMT and BER are not involved in the acquisition of TMZ resistance. Furthermore, in clinical samples, we could not find any correlation between MGMT expression and tumour recurrence (Supplementary Figure S3A, B).

Previous studies have indicated a relationship between deficiencies in MMR components (MSH6, MSH2, MLH1, PMS2) and GBM recurrence [5-7]; however it remains unclear which component is most relevant for the acquisition of TMZ resistance. A decrease in MLH1 expression was observed in all three independent TMZ-resistant cell lines, and attenuation of MLH1 expression consistently modulated TMZ sensitivity in several GBM cell lines (Fig. 1A, B; Table 2). Furthermore, PMS2 protein expression was reduced in these TMZ-resistant cells and was correlated to that of MLH1 (Fig. 1A, B).

We clearly demonstrated that MLH1 or PMS2 knockdown confers TMZ resistance to GBM cells. Interestingly, the protein expression of PMS2 was not correlated with its mRNA expression levels. MLH1 knockdown by siRNA decreased PMS2 protein expression and not PMS2 mRNA expression. Moreover, the expression of PMS2 protein was associated with that of MLH1 protein in several GBM cell lines (Fig. 2A, B). However, the protein expression of MSH2 and beta-catenin was not affected by attenuation of MLH1 expression (Fig. 1D). This would suggest that MLH1 protein expression specifically affects the stability of the PMS2 protein. In contrast, PMS2 knockdown slightly affected the protein expression of MLH1 (Fig. 2C). Previously, Mohd *et al.* showed that PMS2 was stabilized in the presence of MLH1 through heterodimer formation by using an overexpression system [20]. We obtained consistent results indicating that MLH1 expression is responsible for the stability of endogenous PMS2 protein in GBM cells using MLH1 siRNA.

Stark *et al.* have demonstrated that MLH1 expression is significantly reduced in recurrent GBM, and that its expression in initial lesions was an indicator of reduced patient survival [6]. On the other hand, Flesberg *et al.* showed that the expression of MSH6, MSH2, and PMS2 were reduced in recurrent GBM, but that MLH1 expression was not correlated to recurrence [7]. However, in the Felsberg study, 32.6% of the recurrent tumours showed lower levels of MLH1 expression than in the primary tumours. This could mean that a decrease in MutL alpha may occur. We revealed that the suppression of MLH1 expression decreases the expression of PMS2 protein in TMZ-resistant cells and GBM cell lines in *vitro*. However, in our clinical data, though the expression of MLH1 and PMS2 decreases, the alternation of the expression of these genes did not always correlate each other (Fig. 3B). In addition, the protein expression of MSH6 was also decreased in U251/TMZR1 cells (Fig. 1B). These facts suggest that a decrease in MLH1 expression occurs early during the acquisition of TMZ resistance and, accordingly, the expression of PMS2 protein is reduced, although other factors may influence the expression of MMR component. However, the mechanism by which this occurs remains to be elucidated.

There is some new knowledge about the mechanism of chemoresistance in GBM. Urszula et al. revealed that loss of PDCD4 contributes to enhanced chemoresistance in GBM [21]. Swapna et al. revealed that epigenetic regulation of miRNA-211 by MMP-9 give the insensitivity of GBM to radiation and TMZ [22]. It might necessary to examine the function of such molecules in our TMZ-resistant cells. In addition, some studies propose new drugs for GBM treatment [23, 24]. Some clues to overcome TMZ resistance might be revealed by examine the effect of such new drugs against our TMZ-resistance cells.

In summary, we have shown that U251 GBM cells acquire resistance to TMZ by reducing MLH1 expression following destabilization of PMS2 protein that is attenuated by MutL alpha. In addition, a significant reduction in MLH1 and PMS2 expression was observed in recurrent GBM tumours during TMZ administration. Our data suggest that a reduction in MLH1 protein expression leads to PMS2 protein instability, which confers TMZ resistance on GBM cells. This could lead to the recurrence of GBM during the course of TMZ treatment. Evaluating the expression of MLH1 and PMS2 in GBM may therefore provide a useful index for predicting the efficiency of TMZ anti-tumour activity.

## MATERIALS AND METHODS

### Drugs, Reagents and antibodies

The following reagents were purchased from the indicated companies (in brackets): RPMI 1640 (Nikken Biomedical Laboratory, Osaka, Japan); Dulbecco's modified Eagle's medium (DMEM) (Nissui Seiyaku, Tokyo, Japan); foetal calf serum (FCS) (**PAA Laboratories,** Pasching, *Austria*); Coulter DNA Prep Reagents Kit (Beckman Coulter, Inc, Fullerton, CA, USA); Ac-DEVD-MCA (Ac-Asp-Glu-Val-Asp-MCA) (Wako Pure Chemical Industries, Osaka, Japan); MTT (3-(4,5-dimethylthiazol-2-yl)-2,5-diphenyl tetrazolium bromide) (Sigma-Aldrich, St, Louis, MO, USA); SYBR® Premix Ex Taq™ II (Takara, Osaka, Japan); monoclonal antibodies against MLH1, beta-catenin, MGMT, MSH2, GAPDH (glyceraldehyde-3-phosphate dehydrogenase), MSH6, PMS2 and alpha-tubulin (BD Pharmingen, San Diego, CA,USA; Chemicon International, Inc, Temecula, CA, USA; Cell Signaling Technology, Inc., Danvers, MA, USA; Abcam, Cambridge, UK; and Calbiochem, San Diego, CA, USA, respectively).

### Cells and cell culture

Human U251, U373, U105, LN229 GBM cells were cultured in RPMI 1640 containing 10% foetal calf serum and a 1% antibiotic-antimycotic solution (Invitrogen, Carlsbad, CA, USA). To isolate TMZ-resistant cells, U251 cells were cultured in selection medium containing 400 micro-M TMZ and cloned by using the limiting dilution method. Three TMZ-resistant cells were then isolated and named U251/TMZR1, U251/TMZR2 and U251/TMZR3. All cell lines were cultured at 37 °C in a 5% CO_2_-humidified atmosphere.

### RNA interference

MLH1 siRNA were purchased from Sigma-Aldrich. Sequences were: 5ʹ-UCACAAGUAUUCAAGUGAdTdT-3ʹ(Sense oligo# 7134001) and 5ʹ-UCACUUGAAUACUUGUGGAdTdT-3ʹ(Antisense oligo#7134002). The PMS2 siRNA duplexes were based on the coding region of the gene of interest, designed to contain dTdT overhangs, and were obtained from FASMAC. The sequences of the siRNAs were: 5ʹ-CAAUGUUACUCCAGAUAAAdTdT-3ʹ(Sense) and 5ʹ-UUUAUCUGGAGUAACAUUGdTdT-3ʹ(Antisense). Silencer® Negative Control No. 1 siRNA (Ambion, Catalog #: AM4611) was used as the control. One day before transfection, cells were seeded into 6-cm tissue culture dishes at a density of 3×10^5^ cells. Cells were then transfected with siRNA using Lipofectamine 2000 (Invitrogen, Carlsbad, CA, USA) according to the manufacturer's instructions. The cells were transferred to 96-well plates (5×10^2^/well) 24 h after transfection for assay using the MTT colorimetric assay, or were transferred to 6-cm tissue culture dishes 48 h after transfection for real-time PCR and immunoblotting analyses.

### MTT assay of cell survival

Equal numbers of cells (5×10^2^) were inoculated into each well and the cells were treated for 7 days with TMZ, ACNU, MMS, or MNNG before the sensitivity of the cells to each of the administered drugs was measured using a MTT colorimetric assay as described previously [25].

### Caspase-3 activation assay

U251 and U251/TMZR2 cells were seeded into a 6-cm tissue culture dish at a density of 2×10^5^ cells. After treatment with 800 micro-M TMZ for 96 h, the cells were trypsinized, and caspase-3 activation assays were carried out as described previously [26].

### Measurement of apoptotic cells and cell-cycle analysis by flow cytometry

U251 or U251/TMZR2 cells were seeded into 6-cm dishes at a density of 2×10^4^ cells. After overnight incubation, the cells were treated with 800 micro-M TMZ for 24, 48 or 120 h. Measurements of apoptotic cells and cell-cycle analysis were carried out by flow cytometry as described previously [27].

### Methylation-specific PCR

See Supplementary Materials and Methods.

### RNA isolation and cDNA synthesis

Total RNA from the cultured cells was isolated using the TRI reagent (Molecular Research Center, Cincinnati, OH, USA) according to the manufacturer's instructions. RNA (1 micro-g) was reverse-transcribed using the ReverTra Ace kit (Toyobo, Osaka, Japan).

### Reverse transcription–PCR

See Supplementary Materials and Methods..

### Quantitative real-time PCR

The mRNA expression levels of MSH6, MSH2, MLH1and PMS2 were determined by real-time RT-PCR (PRISM 7900HT; Applied Biosystems, Foster City, CA, USA) using SYBR® Premix Ex Taq™ II (Takara, Osaka, Japan) according to the manufacturer's instructions. Human GAPDH was used for normalization. The expression of the target gene was quantified by using the comparative cycle threshold method. Forward and reverse primers respectively were as follows: for MSH6, 5ʹ- AGAGCAATGCAACGTGCAGA-3ʹ and 5ʹ- TTTGGCGGCTACTTCGCCTA-3ʹ; for MSH2, 5ʹ- TTTACCCGGAGGAGAGACTGC-3ʹ and 5ʹ- TGCTCTCCCTTTTTGCCTTTC-3ʹ; for MLH1, p5ʹ- TGTGCTGGCAATCAAGGGAC-3ʹ and 5ʹ- TGTCCACGGTTGAGGCATTG-3ʹ; for PMS2, 5ʹ- ATCGGCGAAGGTTGGAACTC-3ʹ and 5ʹ- CGGATGCCTGCTGAAATGAT-3ʹ; for GAPDH, 5ʹ- TGCACCACCAACTGCTTAG-3ʹ and 5ʹ- GAGGCAGGGATGATGTTC-3ʹ.

### Protein extraction and immunoblotting

The cells were harvested and lysed with RIPA buffer (25 mM Tris-HCl (pH 7.5), 150 mM NaCl, 1% Nonidet P-40 (NP-40), 0.1% SDS, 0.5% sodium deoxycholate, 1 mM p-amidinophenyl methanesulfonyl fluoride hydrochloride (APMSF), and 1 micro-g/mL aprotinin), passed through a 27-gauge needle, and centrifuged at 14000 *g* for 15 min at 4 °C. The supernatant was immediately used or was stored at −80°C until use. Protein concentrations were determined using the Bio-Rad protein assay kit. Cell lysates (100 micro-g protein) were subjected to 7.4% or 9.4% SDS-polyacrylamide gel electrophoresis (SDS-PAGE) and transferred onto membranes as described previously [26]. The blotted membranes were incubated with anti-MGMT (1:1000 dilution), anti-MLH1 (1:2000 dilution), anti-PMS2 (1:1000 dilution), anti-MSH6 (1:500 dilution), anti-MSH2 (1:1000 dilution), anti-beta-catenin (1:1000 dilution) or anti-GAPDH (1:200000 dilution) antibody overnight at 4°C, and each protein was detected as described previously [26].

### Immunohistochemical analysis of patient tumours

Tumour specimens were obtained from nine GBM and two anaplastic astrocytoma (AA) patients. After the first surgery, all patients were treated with TMZ (75 mg/m^2^ daily for 42 days) concurrent with conventional radiation therapy (60 Gy), followed by TMZ (200 mg/m^2^) every 28 days according to the EORTC/NCIC-protocol [3] at the Department of Neurosurgery, Kagoshima University Hospital.

Surgically obtained specimens were fixed in 10% formaldehyde and then embedded in paraffin before being cut into 3 micro-m slices. Microwave antigen retrieval was performed in citrate buffer (pH 6.0) before the samples were incubated with the MLH1 mouse monoclonal antibody (1:100 dilution), PMS2 rabbit monoclonal antibody (1:200 dilution), or the MGMT mouse monoclonal antibody (1:100 dilution) as the primary antibody. The number of cells in three microscopic fields (magnification × 400) was counted independently by two researchers (Y.S and S.Y). Ratios of the positive cells were obtained by dividing the number of immunopositive cells by the total number of cells per field, and are expressed as a percentage. Approval for this study was obtained from the Clinical Study Review Board of Kagoshima University Medical and Dental Hospital.

### Statistical analysis

Statistical comparisons were performed using student's *t* test. For immunohistochemical analysis, statistical comparisons were performed using the paired *t* test. Quantitative data were expressed as the means ± SD. P < 0.05 were considered significant.

## Supplementary Figures


