# Supplementary material for: Reduction of MLH1 and PMS2 confers temozolomide resistance and is associated with recurrence of glioblastoma
Source: Oncotarget. 2013 Oct 14;4(12):2261–70. doi: 10.18632/oncotarget.1302 (PMC3926825; doi:10.18632/oncotarget.1302)
Supplement: Supplementary file 1 [file oncotarget-04-2261-s001.pdf]

Reduction of MLH1 and PMS2 confers temozolomide resistance and is associated with recurrence of glioblastoma - Shinsato et al

Supplementary Materials and Methods

Methylation-specific PCR.

Bisulfite modification was performed using The CpGenome™ DNA Modification Kit (Chemicon International, Inc, Temecula, CA) according to the manufacturer's instructions. The treated DNA was resuspended in 25 micro-L of TE and was immediately stored at -20 °C until use. The DNA methylation status of CpG islands in the *MGMT* promoters was determined by methylation-specific PCR (MSP). For PCR amplification, previously reported specific primers [1] were used. The PCR was run as follows: after a 5 min denaturation at 95 °C, 35 cycles of PCR were carried out, with each cycle comprised of 30 s of denaturing at 94 °C, 30 s of annealing at 60 °C, and 1 min of extension at 72 °C, with an extension at 72 °C for 5 min as the last step. The methylation-negative DNA control was purchased from New England Biolabs (Beverly, Mass). The methylation-positive DNA control sample was made *in vitro* using SssI methylase (New England Biolabs). The PCR products were separated on a 6% agarose gel.

Reverse transcription–PCR

The first-strand cDNA (1 micro-L) resulting from reverse transcription was used for each PCR. The human MGMT primers used were: 5′-CCTGGCTGAATGCCTATTTC-3′ (forward primer) and 5′-AGGGCTGCTAATTGCTGGTA-3′ (reverse primer). Primers for the GAPDH internal control were: 5′-ACCACAGTCCATGCCATCAC-3′ (forward primer); and 5′-TCCACCACCCTGTTGCTGTA-3′ (reverse primer).

Twenty-seven cycles were performed, with each cycle comprised of 98 °C for 10 s for denaturation, 55 °C for 30 s for annealing, and 72 °C for 1 min for extension. MGMT full-length cDNA was used as a positive control.

References

1.Wang J, Sasco AJ, Fu C, Xue H, Guo G, Hua Z, Zhou Q, Jiang Q and Xu B. Aberrant DNA methylation of P16, MGMT, and hMLH1 genes in combination with MTHFR C677T genetic polymorphism in esophageal squamous cell carcinoma. Cancer epidemiology, biomarkers & prevention : a publication of the American Association for Cancer Research, cosponsored by the American Society of Preventive Oncology. 2008; 17(1):118-125.

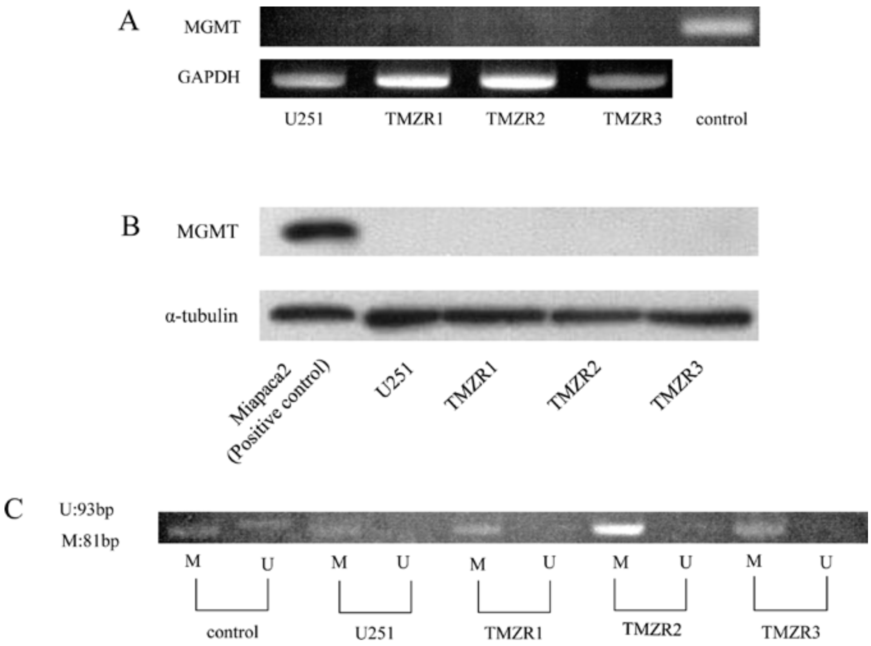

Supplementary Figure S1: MGMT expression in U251 parental and TMZ-resistant cells.(A) The mRNA level of MGMT in U251 and TMZ-resistant cells (U251/TMZR1 ,U251/TMZR2 and U251/TMZR3 cells) was analysed using RT-PCR. GAPDH mRNA was used as a loading control. (B) The protein level of MGMT in U251 and in TMZ-resistant cells was detected by immunoblotting with the anti-MGMT antibody indicated in Materials and Methods. α-tubulin was assayed as a loading control. (C) The methylation status of the *MGMT* promoter of U251, and of the U251/TMZR1 ,U251/TMZR2and U251/TMZR3 TMZ-resistant cells, was analysed using methylation-specific PCR. The methylation-negative DNA control was used for the unmethylated control. The methylated control was made *in vitro* using SssI methylase from the methylation-negative DNA control. U, PCR products amplified by

unmethylated-specific primers; M, PCR products amplified by methylated-specific primers.

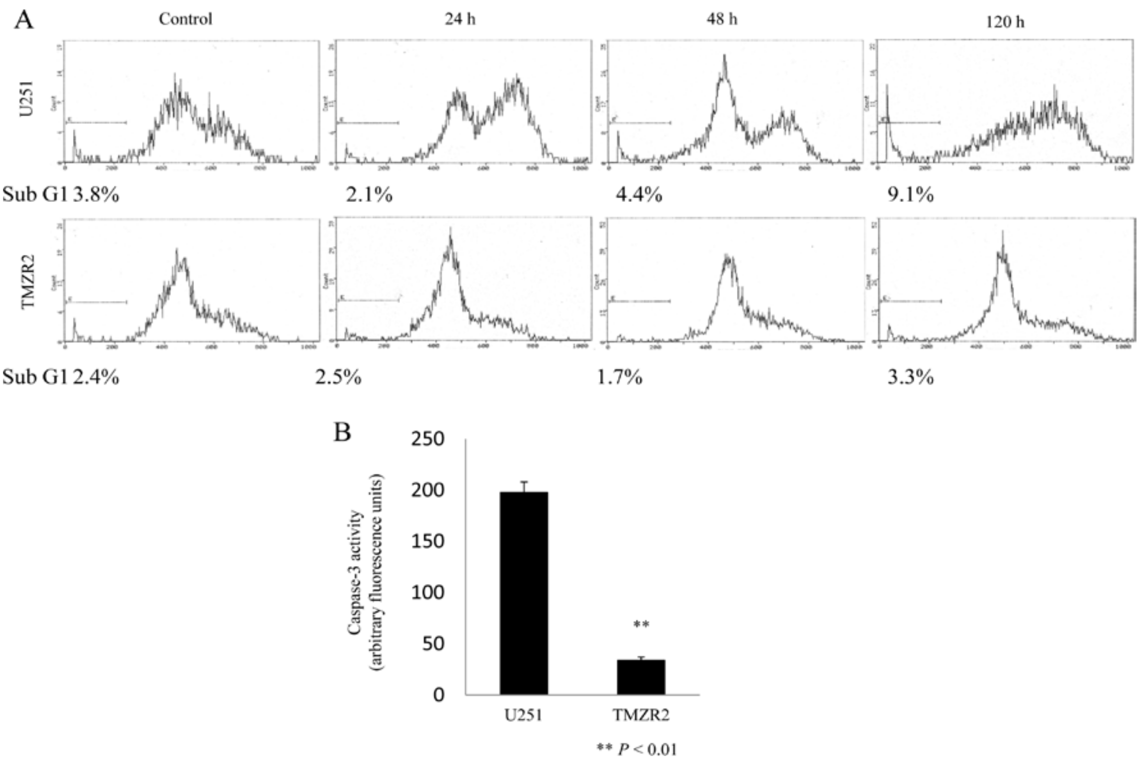

Supplementary Figure S2: Analysis of TMZ induction of G_2/M arrest and apoptosis in U251 and U251/TMZR2 cells. (A) Following exposure of U251 and U251/TMZR2 cells to TMZ (800 micro-M) for 24, 48 or 120 h, cell cycle stages and apoptosis (% of cells in the sub-G_1 stage) were assessed by flow cytometry following propidium iodide staining. The corresponding untreated cells were used as controls. Bar, Sub-G_1 fraction. (B) Following treatment of U251 and U251/TMZR2 cells with 800 micro-M TMZ for 96 h, caspase-3 activity was measured in cell lysates containing equivalent amounts of protein using a specific substrate: Ac-DEVD-MCA. The data are expressed in arbitrary units. Each value represents the mean of three independent experiments. Bars, SD. **, $P < 0.01$.

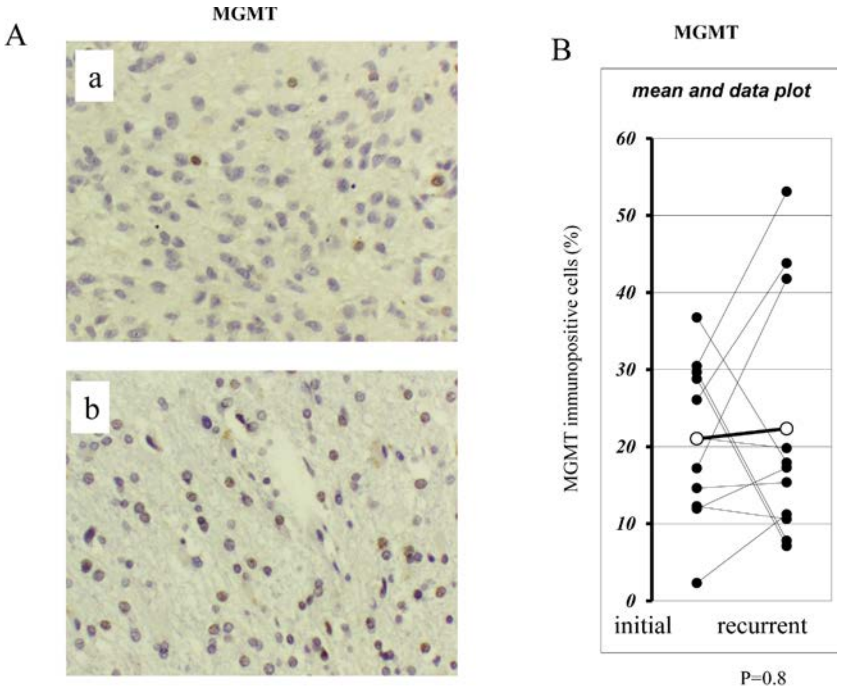

Supplementary Figure S3: Immunohistochemical analysis of the expression of MGMT in recurrent human glioblastomas during administration of TMZ. MGMT protein expression in nine clinical glioblastoma and two anaplastic astrocytoma specimens was analysed by an immunohistochemical method as described in Materials and Methods. The fraction of MGMT-positive cells in each specimen was determined by dividing the number of cells immunopositive for the MGMT antibody by the total number of cells in three microscopic fields (magnification × 400) and was expressed as a percentage. Immunohistochemical staining of initial and recurrent tumours of one representative case, the same as Fig. 3A, is shown. (a) MGMT staining in the initial tumour (2.3 ± 1.2 % positive nuclei) versus (b) in the recurrent tumour (11.2 ± 3.3 % positive nuclei). (B) The percentage of MGMT-immunopositive cells in each clinical initial and recurrent gliobastoma specimen is shown (paired *t*-test, $P = 0.8$).
